# ID 284 - JAV-RARAS: Estudo de Aderência aos Protocolos Clínicos e Diretrizes Terapêuticas (PCDT) em Esclerose Lateral Amiotrófica: comparação entre PCDT e realidade prática em serviço de atenção em doenças raras

**DOI:** 10.5327/2237-9622.2025.v34s1.284

**Published:** 2025-11-25

**Authors:** Camila Correa Azevedo, Myrianne Gilsara Soares e Barbosa, Marilaine Menezes Ferreira, Marcela Mahado, Marcelo Nita, Luana Lopes, Thiago Godoy, Têmis Félix

**Keywords:** *compliance*, consensos médicos, processos assistenciais, gestão clínica

## Abstract

**Introdução:**

O estudo de aderência ao Protocolo Clínico e Diretrizes Terapêuticas (PCDT) faz parte do JAV-RARAS, da Rede Nacional de Doenças Raras (RARAS), centrando-se na avaliação da Jornada Assistencial de Valor (JAV) para pacientes com doenças raras no Brasil. Os PCDT elaborados pelo Ministério da Saúde têm como objetivos padronizar os tratamentos, assegurar eficácia e segurança, otimizar o uso de recursos e auxiliar na tomada de decisões. A coleta foi feita em serviço de atenção em doenças raras localizada em Salvador, Bahia. Este estudo tem como objetivo específico realizar a avaliação da adesão ao tratamento da esclerose lateral amiotrófica (ELA), conforme os parâmetros definidos pelo PCDT. A análise utiliza dados da jornada do paciente coletados para implementar a metodologia (TDABC), permitindo uma compreensão detalhada das interações e do uso de recursos para melhorar a eficácia e a segurança no tratamento de pacientes com ELA.

**Método:**

A avaliação da adesão ao PCDT é fundamentada nos processos assistenciais que foram mapeados no contexto do estudo de TDABC. Inicialmente, realizamos o mapeamento da jornada de cuidados, envolvendo a quantificação do tempo e dos recursos alocados nas fases de diagnóstico, tratamento e acompanhamento. Os dados sobre a jornada do paciente foram coletados por meio de entrevistas e registros fornecidos pelos profissionais de saúde.

**Resultados:**

Os resultados revelam que as atividades na jornada e na alocação de recursos nem sempre estão em conformidade com as diretrizes estabelecidas pelo PCDT. No caso da ELA, o custo direto anual da jornada do paciente, conforme a realidade institucional, é de R$ 36.978,56, enquanto o valor previsto no PCDT é de R$ 42.540,86, ambos com uma maior concentração de recursos direcionados ao tratamento. A distribuição dos recursos ao longo da jornada também apresenta variações significativas: o PCDT aloca R$ 38.227,96 para tratamento, R$ 3.424,00 para diagnóstico e R$ 843,00 para acompanhamento, enquanto o serviço indica uma alocação de R$ 35.207,06, R$ 753,75 e R$ 918,00, respectivamente. Em termos de recursos, o serviço apresenta custo anual de equipamentos de R$ 19.164,10, exames de R$ 744,00, materiais de R$ 89,40, medicamentos de R$ 12.256,56, procedimentos de R$ 1.897,00, e profissionais de R$ 2.827,50. O fluxo descrito no PCDT prevê custos de R$ 3.544,00 para materiais, R$ 810,00 para medicamentos e R$ 35.403,96 para procedimentos, alinhando-se à realidade do centro, com um custo de R$ 885,90 para profissionais. Adicionalmente, o estudo mapeia a origem dos recursos, que também apresentam discrepâncias. No PCDT para ELA, 0,77% dos recursos provém do próprio paciente, 3,02% provêm do serviço, 5,88% da indústria e 90,3% do Sistema Único de Saúde (SUS).

**Conclusão:**

A avaliação da adesão ao tratamento da ELA no âmbito do projeto JAV-RARAS revela sobre a jornada assistencial de pacientes com ELA no serviço. Os dados coletados demonstram que, embora o PCDT vise padronizar e otimizar os cuidados, a realidade observada indica uma discrepância entre os custos reais e os previstos, além de variações na alocação de recursos ao longo do processo assistencial.

